# Automatic object-based spatial selection depends on the distribution of sustained attention

**DOI:** 10.3758/s13414-021-02325-x

**Published:** 2021-06-15

**Authors:** Ema Shamasdin Bidiwala, Miranda Scolari

**Affiliations:** Department of Psychological Sciences, Texas Tech University, MS 2051, Lubbock, TX 79409 USA

**Keywords:** Object-based attention, Space-based attention, Spatial spotlight, Flanker task, Inducer task

## Abstract

Several space-based and object-based attention studies suggest these selection mechanisms may be voluntarily deployed, depending on task parameters and the attentional scope of the observer. Here, we sought to elucidate factors related to involuntary deployment of object-mediated space-based attention through two experiments. Experiment 1 used a modified flanker task where a target and nearby distractor were presented within the same or different object frames, such that an object-based attentional spread should be detrimental to performance. Results showed the presence of a flanker effect with no significant difference in magnitude between grouping conditions, indicating participants may have uniformly used a diffused attentional spotlight regardless of object segmentation. In a second experiment, we manipulated the extent of the observer’s sustained attentional scope via an inducer task to determine whether object-based selection depends on the initial spotlight size. The results revealed object-based effects solely when attention narrowly encompassed the target, but not when it was widened to include the distracting flanker. This suggests the deployment of object-based attention may occur when spatial attention is initially focused narrowly. Because selecting the whole object frame directly interfered with task goals, we conclude that object-based attention may not always fully conform to relevant task goals or operate in a goal-oriented manner. We discuss these results in the context of existing literature while proposing a reconciliation of previously inconsistent findings of object-based selection.

## Introduction

A longstanding goal of visual attention research is to fully understand how sensory input influences selection. A “spotlight” metaphor for covert space-based attention (SBA) that has endured for decades effectively describes how precues can govern the size of selected space and modulate target discriminability (Broadbent, 1982; C. W. Eriksen & Hoffman, 1972; C. W. Eriksen & Rohrbaugh, 1970; Huang et al., 2016; LaBerge, 1983; McMains & Somers, 2004; N. G. Müller et al., 2003; Posner, 1980; Remington & Pierce, 1984; Shulman et al., 1979; Tsal, 1983; Womelsdorf et al., 2006). The use of object contours similarly shows enclosures can govern the shape of selected space (Al-Janabi & Greenberg, 2016; Chen & Cave, 2006, 2008; Egly et al., 1994; Fiebelkorn et al., 2013; Lamy & Egeth, 2002; Moore et al., 1998; Shomstein, 2012). As a result, many investigators have concluded that selection can be driven largely by sensory input, but few studies employed tasks where performance was decidedly hindered by attending the whole cue or object frame. The few that have attempted to dissociate sensory-driven and goal-oriented attention (Castiello & Umiltà, 1990; Huang et al., 2016; Kramer & Jacobson, 1991; Shomstein & Yantis, 2002; Turatto et al., 2000) produced conflicting findings. This raises the question: When sensory input and behavioral goals are misaligned, under what circumstances does the former determine selection?

## Cue size manipulations of covert spatial selection

Researchers have used cue size to manipulate the focus of attention (Burnett et al., 2013; Castiello & Umiltà, 1990; Greenwood & Parasuraman, 1999, 2004; Mizuno et al., 1998; Mounts & Edwards, 2016; Turatto et al., 2000; Yeshurun & Carrasco, 1998; 2008). In many experimental designs, however, using the whole cue is advantageous, as possible target locations are distributed across the full extent of the cued space (C. W. Eriksen & James, 1986; C. W. Eriksen & Yeh, 1985; Greenwood & Parasuraman, 1999, 2004). It is unclear, then, whether cueing effects result from an adherence to task demands via controlled voluntary attention, are driven by bottom-up sensory input, or some combination of both.

To address these questions, Huang et al. (2016) independently manipulated spatial uncertainty and cue size. They found that RT in a detection task predictably and reliably increased with spatial uncertainty. In contrast, the effects of cue size were negligible when spatial uncertainty was held constant. These results implicate a voluntary attention system that controls the size of selected space, where the spatial extent of a cue may only be utilized if it is behaviorally advantageous. However, the results of this study run counter to a small number of studies demonstrating cue size effects even in the absence of target location uncertainty (Castiello & Umiltà, 1990; Turatto et al., 2000). It is thus possible that while introducing spatial uncertainty is a more reliable method for manipulating attentional field size, spatial cue size can nonetheless shape selection under certain circumstances, even when changes in cue size do not confer performance benefits.

One effective approach to modulate attentional field size while maintaining spatial certainty is to attach relevance to the cue that nonetheless elicits a malformed field with regard to specific target identification goals (Goodhew et al., 2016; Goodhew et al., 2017; LaBerge, 1983; Lawrence et al., 2020). During an inducer task, Goodhew and colleagues instructed participants to make shape judgments about an enclosed, centrally positioned frame, serving to produce a stable attentional spotlight of a prescribed size. An interleaved second spatial discrimination task required a focal spotlight at center. Since the sustained spotlight was shaped by the inducer, a performance benefit (small inducer) or cost (large inducer) carried over to the main discrimination task. The size of spatial selection, therefore, may not always conform to immediate task goals, especially when the attentional state is modulated by orthogonal task demands.

## Object-based manipulations of covert spatial selection

An analogous line of research grapples with similar questions regarding the shape of selection. Object-based attention (OBA) is the selection of a group of features or region of space bound into a holistic representation (Scolari et al., 2014). Here, we focus on a distinct subset of object-based studies within the literature that explores if and when spatial selection is governed by object contours (Scholl, 2001); we consider this object-mediated space-based selection. Many paradigms from this line of work resemble those used in the cue size studies. For example, the classic two-rectangle paradigm is commonly used to explore whether cueing one end of a rectangular frame results in selection of the full enclosed space, as assessed by detection performance of targets positioned at either end (Al-Janabi & Greenberg, 2016; Chen & Cave, 2006, 2008; Egly et al., 1994; Fiebelkorn et al., 2013; Lamy & Egeth, 2002; Moore et al., 1998; Shomstein, 2012). Such results are indicative of object-mediated space-based selection.

Early OBA studies often made inferences about an involuntary attentional spread that occurred within object contours, implicating sensory-driven selection. However, most patterns of this sort are similarly consistent with a voluntary account. Much like the cue size effect studies described above, experiments of this nature often failed to adequately differentiate sensory-driven selection from goal-oriented selection. Given that the target could appear—albeit infrequently—in uncued locations within the cued object, selecting the whole object frame increases the likelihood that the upcoming target location is attended compared to only selecting the spatially cued location. As such, object-mediated space-based selection may depend at least in part on task demands rather than purely on stimulus-driven mechanisms (Al-Janabi & Greenberg, 2016; O’Bryan & Scolari, 2021; Shomstein, 2012; Shomstein & Behrmann, 2008; Shomstein & Yantis, 2004, 2006).

Shomstein and Yantis (2002) attempted to dissociate between sensory and goal-driven accounts using a flanker task, where targets are flanked by compatible or incompatible stimuli (B. A. Eriksen & Eriksen, 1974; C. W. Eriksen & Schultz, 1979), in conjunction with an object-based display. The target appeared in a fixed location, and flanking distractors appeared either within or outside of the same object frame. The authors found evidence of distractor interference, indicated by a flanker effect, of similar magnitudes regardless of grouping conditions. Thus, Shomstein and Yantis demonstrated that when the target location is certain, attention may be narrowed to select it without regard for the object frame. Only when the authors introduced an uninformative spatial cue, rendering target location unknown before onset, did OBA effects emerge. Much like Huang et al. (2016), these data suggest that object-based effects are not obligatory but are rather likely dependent on task demands (Scolari et al., 2014).

Notably, however, Kramer and Jacobson (1991) drew the opposite conclusion in a qualitatively different object-based flanker task. Instead of reporting the identity of a distinct target positioned within an object-frame, participants made judgments of a vertical line segment that was either connected to flanking distractor lines (resulting in an object frame) or not. When the target and distractors belonged to a common object, flanker effects were statistically larger, despite perfect target location certainty. This result provides compelling evidence for involuntary object-mediated space-based selection, as individuals could only effectively suppress distractors that were not grouped with the target.

Al-Janabi and Greenberg (2016) differentiated between the two types of displays used by Shomstein and Yantis (2002), and Kramer and Jacobson (1991): the first where target–object integration is weak by virtue of a distinct target appearing within an object frame (e.g., Chen & Cave, 2006, 2008; Egly et al., 1994; Lamy & Egeth, 2002; Shomstein & Yantis, 2002), and second where target–object integration is strong, by virtue of the target being part of the object frame (e.g., Duncan, 1984; Kramer & Jacobson, 1991; Watson & Kramer, 1999). Following a series of experiments, Al-Janabi and Greenberg concluded although object-based effects are rather robust in strongly integrated displays, they are much less so when integration is weak. Pertinent to the current study, only when spatial attention was initially focused via an informative, peripheral cue did weakly integrated displays elicit object-based selection. Notably, these types of displays are most reminiscent of space-based cue size studies.

Ultimately, Al-Janabi and Greenberg (2016) framed their results as supporting a voluntary attention account (Lamy & Egeth, 2002; Shomstein, 2012; Shomstein & Behrmann, 2008; Shomstein & Yantis, 2002, 2004). Object contours may be utilized to guide voluntary attentional shifts between multiple possible target locations—a necessity when target position can deviate from the cued location, and the spatial attentional field does not encompass all relevant regions. However, the authors used task designs with at least some level of target uncertainty, such that whole object selection was again largely consistent with—and never antithetical to—task goals.

Because Al-Janabi and Greenberg (2016) did not test OBA effects in cued displays with perfect target certainty, the possibility remains that a similar relationship with spatial attentional scope may explain the divergent pattern of results between Kramer and Jacobson (1991), and Shomstein and Yantis (2002). The absent OBA effect in the latter could be due to (1) low target–object integration and (2) an initially diffuse attentional field, as Shomstein and Yantis did not include a spatial cue to direct attention to one portion of the object frame when the target position was always known in advance. If OBA effects emerge following initially focused attentional spotlights even when target position is certain, this would indicate that object-mediated space-based selection does not uniformly conform to goal-driven accounts.

## Present study

The present study combines experimental principles from aforementioned cue size and object-mediated space-based attention studies to investigate whether an involuntary spread of attention across object contours depends on the spatial extent of sustained attention. Experiment 1 employed a modified flanker task where two peripheral rings contained a target and distractor. To elicit a single object percept on certain blocks, the rings were grouped with a connector line (Behrmann & Tipper, 1994; Kramer & Jacobson, 1991; Scholl et al., 2001; Tipper & Behrmann, 1996). In this scenario, attending to the full object contours results in selection of the distracting flankers, while perfect target certainty eliminates the need for any attentional shifts across relevant locations. Despite several task design differences, we replicate the findings of Shomstein and Yantis (2002).

Next, we tested the prediction that a focused attentional state may lead to object-based selection even with high target certainty. In Experiment 2, we added an inducer task to manipulate the spatial scope of sustained attention (Goodhew et al., 2016; Goodhew et al., 2017) so that it either only encompassed the expected target location in the flanker task (focal, small inducer), or both the target and distractor locations (diffuse, large inducer). This time, we replicate Al-Janabi and Greenberg (2016), in which OBA effects are restricted to periods of focused attention. Given that such OBA effects are decidedly detrimental to task performance, we discuss the results in the context of competing voluntary and involuntary models of object-mediated spatial selection.

## Experiment 1

### Methods

#### Participants

Forty-one participants with normal or corrected-to-normal vision were recruited through the Texas Tech University Research Participation System (SONA) for a 1-hr experiment in exchange for partial course credit. We elected to recruit a relatively large sample size compared with similar behavioral studies (among several recent studies cited in the Introduction, range = 8–41, mean *N* = 29.7; Al-Janabi & Greenberg, 2016; Goodhew et al., 2017; Goodhew et al., 2016; Huang et al., 2016; Lawrence et al., 2020) to accommodate possible individual differences given the challenging flanker task and our predictions regarding an interaction between within-subject conditions. Prior to the experiment, each participant gave written informed consent in accordance with the Institutional Review Board and the Declaration of Helsinki requirements.

#### Materials and stimuli

Stimuli were generated in MATLAB 2016b (Mathworks, Natick, MA) using Psychtoolbox 3 (Kleiner et al., 2007), and displayed on a high resolution (1,920 × 1,080 pixels) color monitor (BenQ XL24301) at a frame rate of 100 Hz. Stimuli included two unfilled black rings presented together in one of four quadrants, each with a diameter of 3° and horizontally spaced 1° apart. The ring closest to the central 0.25° black fixation spot (vertically and horizontally offset from fixation by 4°) always demarcated the upcoming target location and the more peripheral ring (offset from fixation by 4° vertically and 8° horizontally) demarcated the distractor location. Both targets and distractors were composed of four possible rotated letter *T*s (Arial, font size 30 pts), presented at 0°, 90°, 180°, or 270°. One *T* stimulus was centrally presented within each of the object rings. The rings were either connected by a black horizontal line (6-pixel width) or not, depending on the current block (see procedure below).

#### Procedure

We designed a modified flanker task (B. A. Eriksen & Eriksen, 1974; C. W Eriksen & Schultz, 1979) in an attempt to manipulate the spatial extent of selective attention via object contours. This experiment investigated whether the spatial extent of covert attention, as measured by a behavioral flanker effect, would depend on stimulus properties that were contradictory with behavioral goals, resulting in significant object-based effects.

#### Main experiment

**Fig. 1:**
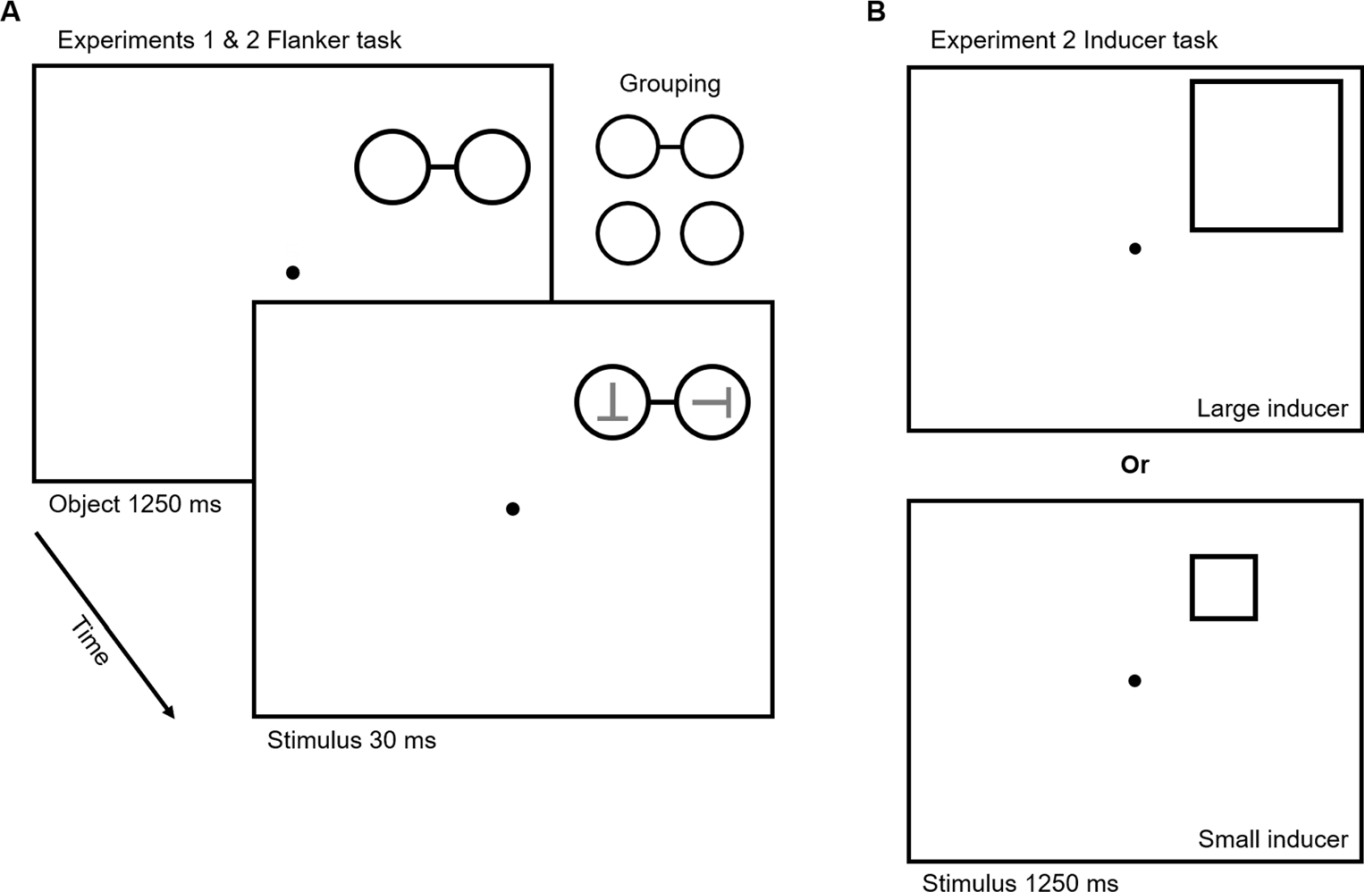
Illustration of the flanker task (**a**) used in both experiments. Two unfilled rings were either presented as separate objects (ungrouped) or as a single object by inserting a connecting line (grouped). The target always appeared in the ring closest to fixation. In this example, and incompatible distractor trial is presented. See main text for design differences between Experiments 1 and 2. Illustration of the inducer task (**b**) used in Experiment 2. Participants judged whether the unfilled shape was a square or rectangle. For each block, participants either completed the task with a large (top) inducer or small (bottom) inducer. Illustrations are not drawn to scale

Each participant was comfortably seated 60 cm from the stimulus presentation monitor in a dark room with the experimenter. During instructions, participants were explicitly told that the target would always appear in the ring closest to fixation (relevant ring) and to ignore the peripheral ring (irrelevant ring). For the first half of the first block (24 trials), the experimenter pointed to the location of the relevant ring to ensure participants understood which item to attend. (See Fig. 1a for an illustration of the experimental events.)

Each trial began with a central black fixation spot (presented alone for 10 s on the first trial of each block and 2 s for each trial thereafter). Next, two rings onset in one of four quadrants (randomly selected on each trial). On alternating sets of two blocks each, the rings were connected by a solid black line to elicit a percept of a single barbell object (grouped condition); the order of grouped and ungrouped blocks was counterbalanced across participants. Consistent with other, similar OBA study designs (Al-Janabi & Greenberg, 2016; Egly et al., 1994; Shomstein & Yantis, 2002), the object display remained on-screen for a 1,250-ms delay before target onset. This duration was expected to be sufficient for participants to identify and direct top-down attention to just the relevant ring, to the extent possible. The target and distractor (if present) next onset within the cue rings for a 30-ms presentation duration, after which the whole display disappeared. The identities of the target and distractor were independently selected on each trial, with the constraint that on one-third of all trials the distractor would match the target (compatible distractor), on one-third of trials it would mismatch the target (incompatible distractor), and on the remaining one-third of trials, only the target was presented (no distractor; baseline condition). Upon stimulus offset, participants were given 2,000 ms to make an accurate-as-possible button-press response using the arrow keys to indicate the orientation of the target *T*. Mean accuracy for each block was presented at the end, at which point participants were invited to take a short break before starting the next block. Participants completed a total of eight blocks of 48 trials each.

#### Eye-tracking

Participants were instructed to maintain fixation for the full length of each trial and to avoid blinking until after response selection. To verify compliance, an experimenter monitored eye position in real time using a video-based eye tracker in chin-rest-free mode (EyeLink 1000 Plus system, SR Research, Ontario, Canada) positioned approximately 55 cm from the participant. Eye data was collected from the right eye at a sampling rate of 500 Hz. Prior to the experiment, the tracker was calibrated using 9 points presented in a random sequence, and eye position was validated to ensure an average prediction error of <1°. In the event of an unwarranted eye movement outside of a prescribed 2° interest area around fixation, the experimenter prompted the participant to return gaze to the center dot.

#### Analyses

##### Exclusion criteria

Off-line, participants’ eye-tracking data were analyzed to ensure proper fixation for a fixed interest period beginning with object onset and ending with response selection. Trials were marked as failed fixations if eye gaze moved outside of the central 2° interest area at any point during the interest period and were removed from further analyses.

Any participants who performed at chance (25% correct) were also marked for removal from further analyses. Finally, trials in which no response was recorded were removed from analyses.

##### Behavioral measures and statistical analyses

Here, we are interested in the effects of irrelevant flankers on perceptual sensitivity rather than response conflict or decision time. Consistent with these goals, participants were instructed to respond as accurately as possible, and we used percentage correct as our main behavioral measure of interest. We calculated participants’ accuracy for each cell in our 2 (grouping condition) × 3 (distractor type) within-subject design. Although we are primarily interested in accuracy, we secondarily report reaction times (RTs) for correct trials only in the same manner as percentage correct. This allows us to verify that any effects we observe in the former cannot be attributed to a speed–accuracy trade-off.

To estimate flanker effects, we compared the no distractor and compatible distractor trials to the incompatible distractor trials, where accuracy is expected to be significantly lower if the attentional spotlight includes the irrelevant ring. Furthermore, we reasoned that if grouping relevant and irrelevant locations together in a common object causes attention to involuntarily spread to the distractor, then flanker effects should be greater in the grouped condition. We thus looked for a possible interaction between grouping condition and distractor type.

We used repeated-measures analyses of variance (ANOVAs) to analyze the accuracy and RT data. We report effect sizes as partial eta squared (${\eta}_p^2$) for all ANOVAs (Hentschke, 2020), where values of 0.01, 0.09, and 0.25 correspond to small, medium, and large effects, respectively. For all *t* tests, we report Hedges’s *g* using the formula: (*M*_1_ − *M*_2_) / *SD*_*within*_, which is closely related to Cohen’s *d* and may be interpreted using the same general guide (small = 0.2, medium = 0.5, large = 0.8; Cohen, 1988; Hedges, 1981; Kline, 2004).

### Results and discussion

#### Exclusions

On average, participants maintained fixation for the duration of the interest period on 92.2% (*SD* = 14.1%) of all trials. See Table 1 for the average percentage of proper fixations for each cell in the 2 (grouped condition) × 3 (distractor type) within-subject design. One participant was removed from analyses due to chance performance on the task. Therefore, all data reported here include the remaining 40 participants.

**Table 1:** Mean percentage of fixations

Grouping	None	Compatible	Incompatible
Grouped			
Exp 1	91.8% (16.8%)	91.3% (17.3%)	90.9% (17.3%)
Exp 2: Large		78.8% (23.1%)	79.6 (23.5%)
Exp 2: Small		78.8% (26.5%)	77.3% (25.9%)
Ungrouped			
Exp 1	93.1% (12.7%)	92% (13.5%)	94.1% (11.2%)
Exp 2: Large		80.5% (20.3%)	80.3% (21%)
Exp 2: Small		77.7% (27.3%)	78.4% (24%)

Note: Mean percentage (standard deviation in parentheses) of proper fixations on the flanker task for each grouping by distractor condition for Experiments 1 and 2. For Experiment 2, the labels “large” and “small” refer to the inducer size

#### Behavioral results

##### Accuracy

A 2 (grouping condition) × 3 (distractor type) repeated-measures ANOVA revealed a significant main effect for distractor type, *F*(2, 78) = 28.1, *p* < .0001, ${\eta}_p^2$ = 0.42. Across grouping conditions, performance was matched between the no distractor (*M* = 67%, *SD =* 14%) and compatible distractor (*M* = 67.8%, *SD* = 14%) conditions, *t*(39) = 0.95, *p* = .35, *g* = 0.056, and significantly lower in the incompatible distractor condition (*M* = 59.3%, *SD* = 15.6%; incompatible distractor vs. mean of other two conditions), *t*(39) = 6.28, *p* < .0001, *g* = 0.55. Thus, the flanking incompatible distractor hindered performance as expected, while a compatible distractor did not provide a detectable perceptual benefit.

**Fig. 2:**
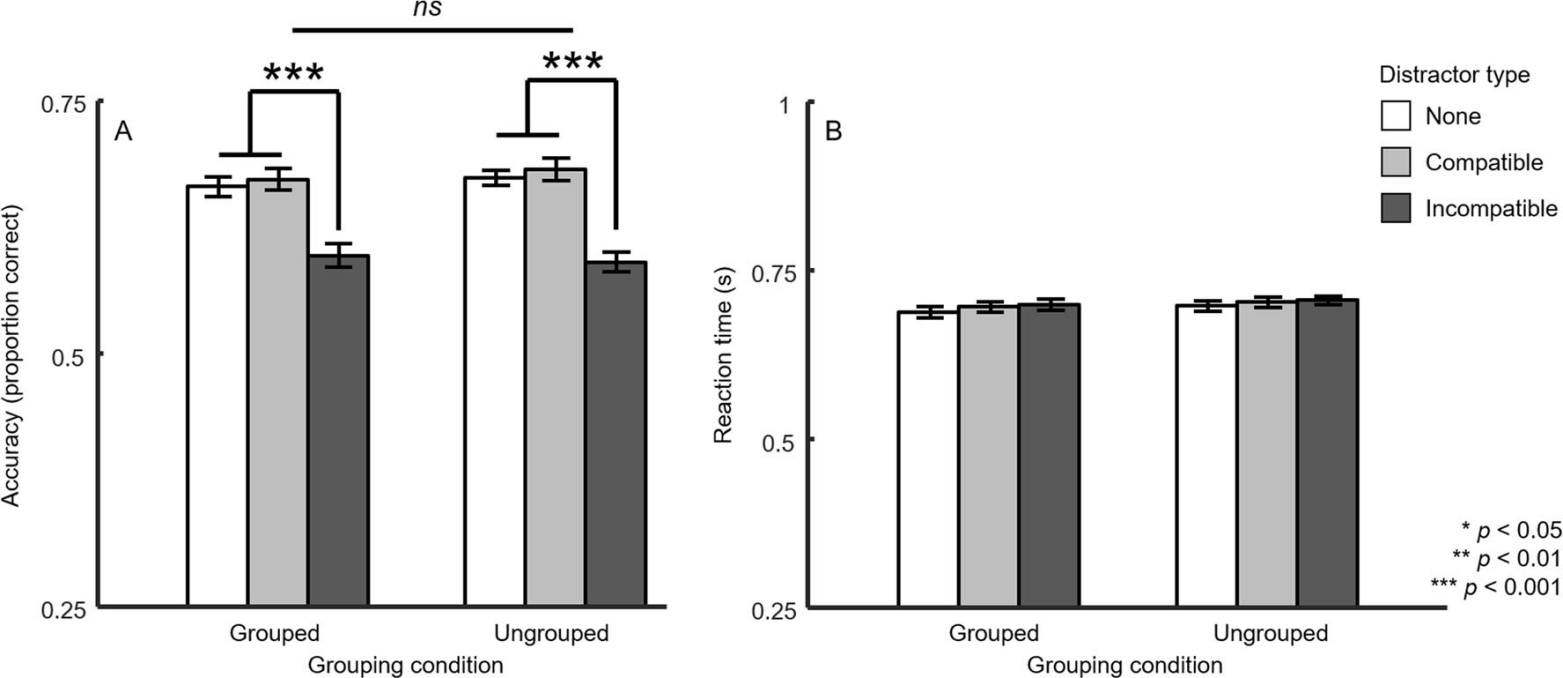
Results from the flanker task in Experiment 1. Performance accuracy (**a**) and reaction time (**b**) are plotted for each distractor type and grouping condition. Mean reaction times (RTs) were calculated for correct trials only. Error bars reflect ±1 within-subject *SEM*

This pattern was observed regardless of whether the relevant and irrelevant rings were grouped together (no distractor: *M* = 66.4%, *SD* = 15.1%; compatible distractor: *M* = 67.2%, *SD* = 14.7%; incompatible distractor: *M* = 59.6%, *SD* = 16.1%), *F*(2, 78) = 12.80, *p* < .0001, ${\eta}_p^2$ = 0.25, or not (no distractor: *M* = 67.3%, *SD* = 14.4%; compatible distractor: *M* = 68.1%, *SD* = 15.3%; incompatible distractor: *M* = 59%, *SD* = 16.3%), *F*(2, 78) = 24.81, *p* < .0001, ${\eta}_p^2$ = 0.39. Furthermore, there was no detectable main effect for grouping condition, *F*(1, 39) = 0.15, *p* = 0.70, ${\eta}_p^2$ = 0.004, nor an interaction, *F*(2, 78) = 0.45, *p* = 0.64, ${\eta}_p^2$ = 0.012 (see Fig. 2a).

##### Reaction time

The RT data suggests the flanker effects described above were not the result of a speed–accuracy trade-off. Mean RTs were statistically well matched for no distractor (*M* = 692 ms, *SD* = 119 ms), compatible distractor (*M* = 699 ms, *SD* = 125 ms), and incompatible distractor trials (*M* = 703 ms, *SD* = 130 ms; see Fig. 2b). A 2 × 3 repeated-measures ANOVA produced no main effects: grouping condition, *F*(1, 39) = 0.43, *p* = .52, ${\eta}_p^2$ = 0.011; distractor type, *F*(2, 78) = 1.07, *p* = .35, ${\eta}_p^2$ = 0.027, and no significant interaction, *F*(2, 78) = .033, *p* = .97, ${\eta}_p^2$ = 0.0008. Furthermore, RTs across distractor types were not significantly different for either the grouped, *F*(2, 78) = 0.77, *p* = .47, ${\eta}_p^2$ = 0.019, or ungrouped, *F*(2, 78) = 0.43, *p* = .65, ${\eta}_p^2$ = 0.011, conditions.

The results of Experiment 1 demonstrate that our experimental design produces strong and reliable flanker effects in accuracy. However, the effect was matched across grouping conditions, indicating that combining the relevant and irrelevant rings into a common object did not elicit an involuntary attentional spread above and beyond the ungrouped condition. This finding may not be surprising, given several experimental parameters that were explicitly put into place to discourage voluntary whole object selection. First, the location of the target was provided with perfect certainty on every trial, and the experimenter even pointed to the relevant ring for the first half of the first block to dispel any confusion. The grouping conditions were also blocked to avoid possible trial-by-trial switches between adaptive selection of structured space (in the ungrouped condition) and adaptive suppression of structured space (in the grouped condition) that could lead to error or fatigue. Finally, should participants transiently select both peripheral rings upon onset, the 1,250-ms presentation duration should have been sufficiently long for participants to voluntarily restrict attention to the relevant ring and suppress input from the irrelevant ring to the extent possible before target onset (Shomstein & Yantis, 2004). The result is also consistent with previous work (Lamy & Egeth, 2002; Shomstein & Yantis, 2002), where significant flanker effects of statistically matched sizes were observed for grouped and ungrouped target and distractor displays. Al-Janabi and Greenberg (2016) similarly failed to detect object-based attention effects in a modified two-rectangle task (Egly et al., 1994) when the target appeared within a precued object (low target–object integration).

Nonetheless, the strong flanker effects (averaging 8.1%) in both grouping conditions indicates that participants failed to adequately suppress the distractor at the irrelevant location. Rather than participants appropriately restricting attention to the relevant location, the attentional spotlight(s) (McMains & Somers, 2004) may have included all display elements regardless of grouping.

This conclusion leaves open at least two possible explanations. First, undue interference from the flanking distractor may be the product of physiological or other low-level factors limiting the spatial resolution of attention, such that the spotlight is maximally focused across conditions. In the presence of sufficient spatial crowding, a target is not fully resolvable during stable fixation (Bouma, 1970; Pelli et al., 2004). Although attention can ameliorate distractor interference, it may have little or no effect on the size of the area where stimulus merging can happen, also referred to as the integration field (Pelli et al., 2004; Scolari et al., 2007; Shiu & Pashler, 1994). Based on this explanation, participants generally deployed top-down attention in an ideal manner, but the spatial resolution limits of the visual system precluded complete distractor suppression. Distractors in the present experiment were positioned just far enough from the peripheral target to be outside the expected integration field by traditional estimates (Bouma, 1970). Notably, though, highly similar stimulus sets like the oriented *T*s used here may elicit greater target–distractor integration than easily distinguishable stimulus sets (Scolari & Awh, 2019), especially when coupled with short presentation times of under 75-ms, like we used here (Tripathy et al., 2014).

Still, unlike spatial attention, other stimulus-driven factors, which help distinguish relevant and irrelevant input, have been shown to reduce the size of the integration field (Scolari et al., 2007). We therefore consider a second possibility: that participants’ attentional spotlights were unduly wide and/or attentional suppression was inadequate (Barras & Kerzel, 2017), as shaped by the entire extent of the object display. In this case, participants may have attended to both the relevant and irrelevant rings, either treating them as a single entity (Chen & Cave, 2006) or deploying two concurrent spotlights (Awh & Pashler, 2000; McMains & Somers, 2004), or failed to suppress input at the irrelevant location, regardless of grouping conditions and task instructions.

To tease apart these possible explanations, we designed a new experiment to predictably manipulate the size of the attentional spotlight while participants engaged in the flanker task. Here, participants completed an inducer task for the majority of trials in which they judged the shape of a stimulus frame. For half the blocks, the frame matched the spatial position and extent of only the relevant ring (small inducer) to elicit a focused attentional spotlight. For the remaining half, a larger frame extended to the most peripheral edge of the irrelevant ring (large inducer) to elicit a diffuse attentional spotlight. Following the logic of Goodhew and colleagues (Goodhew et al., 2016; Goodhew et al., 2017), we reasoned the high frequency, fixed-size inducer trials would produce a stable attentional state that carried over to the less frequent flanker trials. Using this design, we can investigate whether the effect of grouping condition depends on the initial size and spatial scope of the attentional spotlight.

## Experiment 2

We introduced an inducer task in Experiment 2 to manipulate the size and shape of a sustained attentional spotlight. Here, we intermixed trials from the same flanker task described in Experiment 1 with trials from a second task designed to induce either a sustained diffuse or focal spread of attention. If the behavioral patterns depend on the inducer task, we may be able to infer an involuntary spatial spread of attention that is governed by object contours under specific sustained attention conditions (Al-Janabi & Greenberg, 2016).

### Methods

#### Participants

To closely match the sample size of the first experiment as well as several other recent inducer task studies (Goodhew et al., 2016; Goodhew et al., 2017; Lawrence et al., 2020), 42 new participants with normal or corrected-to-normal vision were recruited through the Texas Tech University Research Participation System (SONA) for a 1.5-hr session in exchange for partial course credit. Prior to the experiment, each participant gave written informed consent in accordance with the Institutional Review Board and the Declaration of Helsinki requirements.

#### Materials and stimuli

The apparatus and script to generate stimuli for the flanker task were largely identical to Experiment 1, with the following exceptions. Stimulus locations were restricted to the upper two quadrants to limit the frequency of between-trial attentional shifts and to maximize the carryover effects from the intermixed inducer task (which was also restricted to the upper two quadrants). Given that the no distractor condition did not differ from the congruent distractor condition in the first experiment, this condition was also removed.

Stimuli for the inducer task included two sets of black framed rectangles and squares positioned in the same locations as the flanker stimuli, where each set elicited a diffused or focal attentional spread. A large square (7^o^) positioned in either the upper left or right quadrant occupied the same horizontal space as both rings in the flanker task, while a small square (3^o^) in either quadrant occupied the same space as the relevant ring. Paired rectangles were created by increasing either the height or width of the square by 8% (this change was set to 10% for the first participant). To ensure that squares and rectangles could not be differentiated simply by monitoring the absolute position of the edges closest to fixation and instead required attending to the full aspect ratio, these adjustments were only applied to the top horizontal edge or the vertical edge furthest in the periphery, respectively.

#### Procedure

##### Main experiment

Each participant was seated 60 cm from the stimulus presentation monitor in a dark room with the experimenter. Two tasks were randomly intermixed within each of 8 blocks of 96 trials, where the flanker task described in Experiment 1 made up one-third of the total trials and the new inducer task made up the remaining two-thirds of all trials. Regardless of task, each trial began with a 2-s fixation (with the exception of the first trial of each block where the fixation period was 10 s). Therefore, participants did not know which task they would be performing until stimulus onset. Participants used their left hands for the inducer task responses and their right hands for the flanker task responses (see below) and were instructed to prepare to respond to either trial type.

##### Flanker task

The procedure of the flanker task largely matched that of Experiment 1 with the no distractor condition removed. Distractor type (compatible vs. incompatible), object location (upper left vs. upper right quadrant), and target identity (0°, 90°, 180°, or 270° rotation) were randomly selected on each trial, with the constraint that each member of the set was equally represented across all trials. Due to a small programming error introduced in this experiment, distractor type and object location were fully correlated with each other within all grouping conditions; however, we do not suspect that participants recognized this relationship given their performance on the flanker task (see Results and Discussion section). All other conditions were properly counterbalanced.

##### Inducer task

See Fig. 1b for an illustration of the inducer task. The goal of this task was to elicit a sustained distribution of focal or diffused spatial attention that should carry over to the intermixed, less frequent flanker trials. Following initial fixation on inducer trials, either a black framed square or rectangle (randomly selected on each trial with the constraint that each occurred on half of all trials) appeared in one of the two upper quadrants for 1,250 ms (matching the length of the object display presentation in the flanker task; see Experiment 1 Methods). Participants were instructed to report the shape by pressing the “S” key on the keyboard to indicate square and the “D” key to indicate rectangle within a 2,000-ms response window following inducer offset. In the event of a rectangle, either height or width adjustments were randomly selected on each trial, with the expectation that participants would need to distribute attention across the full extent of the object to make an accurate shape judgment.

All participants were exposed to both large and small inducers to elicit a diffuse and focal spread of attention. Inducer size was held fixed for each half of the experiment (four blocks), with the order alternated across participants, to ensure stable attentional deployment. Importantly, the blocking order for the grouping conditions in the flanker task matched that of Experiment 1, such that participants completed two blocks of each inducer size paired with each flanker grouping condition.

#### Eye-tracking

As in Experiment 1, participants were instructed to maintain fixation throughout each trial and prompted by the experimenter to return gaze to fixation if it was broken. Identical procedures to Experiment 1 were used for eye tracking, except the interest area around fixation was increased to 3° × 3°.

#### Analyses

##### Exclusion criteria

As in Experiment 1, participants’ eye-tracking data were analyzed off-line to ensure proper fixation for a prescribed interest period. We conservatively used a more truncated period, beginning with object display onset and ending with stimulus display offset/response window onset to eliminate failed fixations that do not affect target processing. Trials with failed fixations during this interest period were removed from further analyses.

Any participants who performed at chance on either task (25% correct in the flanker task; 50% correct in the inducer task) were also marked for removal from further analyses. This was critically important for the inducer task, where proper engagement with the task is necessary to produce changes in the shape and extent of sustained spatial attention. Finally, trials with no responses recorded were removed from analyses.

#### Behavioral measures and statistical analyses

We calculated mean accuracy for the inducer task to ensure participants were performing equally well regardless of inducer size. The measures of interest for the flanker task matched those described in Experiment 1. Here, we calculated participants’ accuracy and RT for each cell in our 2 (grouping condition) × 2 (distractor type) within-subject design for focal and diffuse spotlight blocks separately. Furthermore, we investigated whether the magnitude of flanker effects (congruent − incongruent percentage correct) depended on an interaction between the grouping and inducer conditions. As in Experiment 1, we analyzed the data using a repeated-measures ANOVA, and report effect sizes using partial eta squared and Hedges’s *g*.

To determine whether either inducer size elicited flanker effect patterns across grouping conditions that differed from the no-inducer blocks of Experiment 1, we conducted two mixed-design ANOVAs, with inducer condition (large or small vs. absent) as a between-subjects variable and grouping condition (grouped vs. ungrouped) as a within-subjects variable. Here, we define flanker effects as described in the previous paragraph for both experiments, thus discarding the no distractor trials of Experiment 1. The interaction term from each ANOVA is of key interest.

Finally, we conducted two Pearson correlations to assess the relationship between performance on each inducer task (large and small) and automatic object-based selection on the intermixed flanker task. We defined object-based selection on a subject-by-subject basis as the difference in the size of the flanker effect between grouping conditions, or:

$$ {\mathrm{Grouped}}_{\left(\mathrm{Compatible}\kern0.34em \mathrm{distractor}-\mathrm{Incompatible}\kern0.34em \mathrm{distractor}\right)}-{\mathrm{Ungrouped}}_{\left(\mathrm{Compatible}\kern0.34em \mathrm{distractor}-\mathrm{Incompatible}\kern0.34em \mathrm{distractor}\right)}, $$

where larger values indicate object-based selection effects of greater magnitudes.

### Results and discussion

#### Exclusions

On average, participants maintained fixation for the duration of the interest period on 93% (*SD* = 7%) for all trials (across both inducer and flanker tasks). Successful fixation was considerably lower on flanker trials (*M* = 79%, *SD* = 21.4%), potentially a byproduct of infrequent task switching inherent in the design. However, failed fixation rates did not differ across condition cells, *F*(7, 296) = 0.36, *p* = .92, ${\eta}_p^2$ = 0.0097, and all trials in which they occurred were removed from analyses. See Table 1 for the average percentage of proper fixations during the flanker task for each cell in the 2 (grouped condition) × 2 (distractor type) within-subject design.

Four participants were removed from analyses due to chance performance on the inducer task. All data reported here, then, is from the remaining 38 participants.

#### Behavioral results

##### Inducer task: Accuracy

The inducer task was made intentionally challenging to encourage attentional deployment across the full extent of the rectangular frame to accurately judge its shape (square or rectangle), and the accuracy rates bore this out. Across the large and small inducer trials, participants averaged 64.3% correct (*SD* = 6.6%). Importantly, performance was statistically matched across inducer sizes (large inducer: *M* = 62.5%, *SD* = 9.7%; small inducer: *M* = 66.1%, *SD* = 10.7%), *t*(37) = 1.43, *p* = 0.16, *g* = 0.35.

##### Flanker task

Our goal was to determine whether object-based attention effects in our flanker task depend on the sustained attentional state of the observer. To this end, we expected the inducer task to elicit either a diffuse or focal spotlight that extends to the flanker task. If involuntary whole object selection is modulated by the shape and size of sustained attention, we may see a difference in the magnitude of the flanker effect based on grouping condition with the requisite attentional spotlight.

We submitted the accuracy flanker effect (congruent − incongruent) to a 2 (inducer size) × 2 (grouping condition) repeated-measures ANOVA to determine whether the patterns associated with diffuse and focal attentional states differed significantly from each other. There was no reliable main effect for inducer size, *F*(1, 37) = 0.07, *p* = .79, ${\eta}_p^2$ = 0.0019, nor for grouping condition, *F*(1, 37) = 0.16, *p* = .69, ${\eta}_p^2$ = 0.0043. However, we observed a significant cross-over interaction, *F*(1, 37) = 6.41, *p* = .016, ${\eta}_p^2$ = 0.15, such that the grouping effect depended on the sustained attentional state of the observer.

In contrast, the RT patterns did not reliably differ or interact: no inducer size main effect, *F*(1, 35) = 2.39, *p* = .13, ${\eta}_p^2$ = 0.064; no grouping condition main effect, *F*(1, 35) = 0.13, *p* = .72, ${\eta}_p^2$ = 0.0036; no interaction, *F*(1, 35) = 0.024, *p* = .88, ${\eta}_p^2$ = 0.00067.

To unpack the interaction observed in accuracy, we next investigated the effect of grouping condition on flanker task performance for each attentional spotlight size separately.

#### Diffuse spotlight (large inducer blocks)

##### Accuracy

**Fig. 3:**
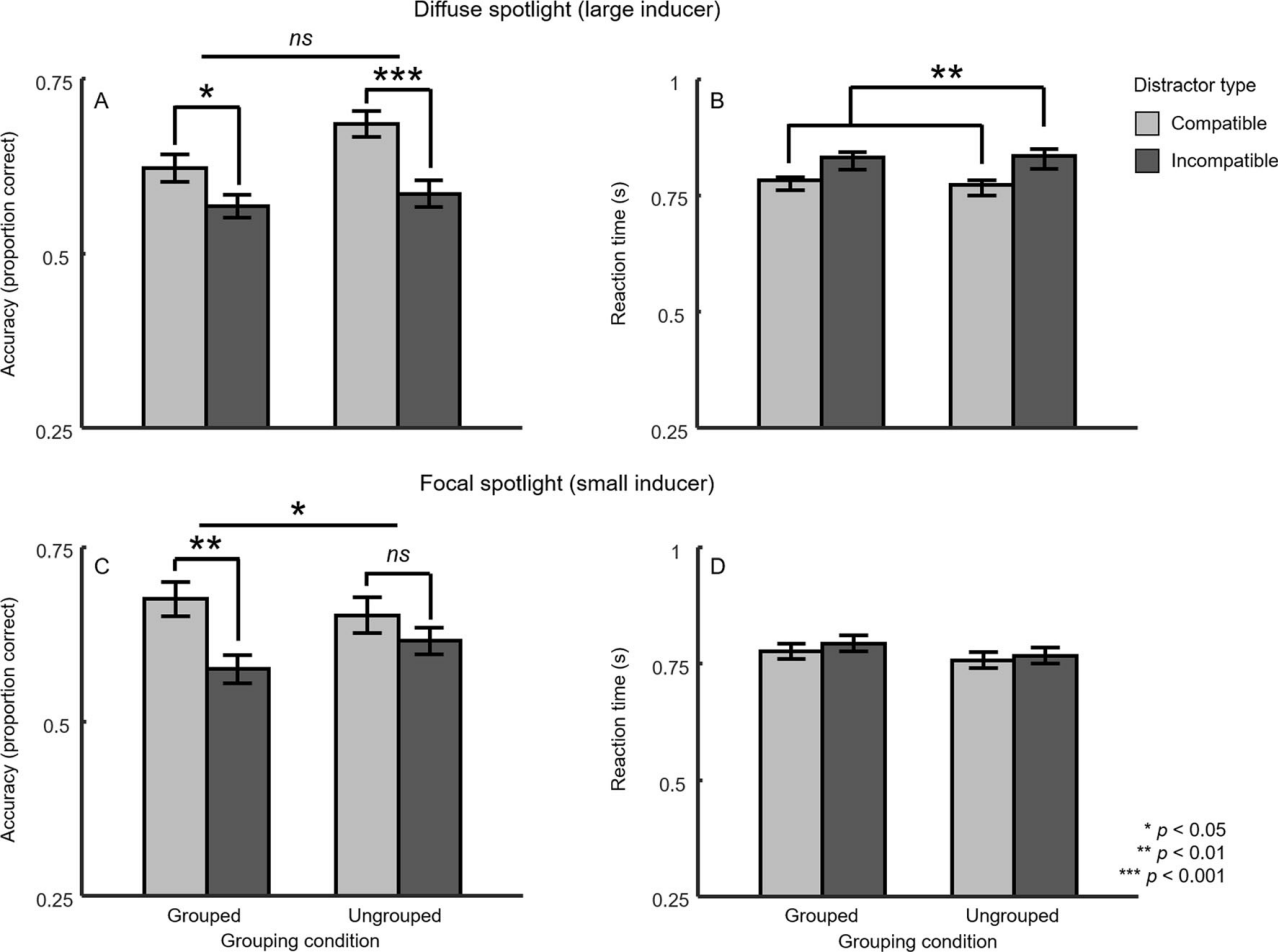
Results from the flanker task in Experiment 2 as a function of the intermixed inducer task. Performance accuracy (**a**) and reaction time (**b**) are plotted for each distractor type and grouping condition when participants were induced to maintain a diffuse attentional spotlight. Performance accuracy (**c**) and reaction time (**d**) are plotted for each distractor type and grouping condition when participants were induced to maintain a focused attentional spotlight. Error bars reflect ±1 within-subject *SEM*

For blocks where diffuse attention was elicited via large inducers, a significant flanker effect emerged in accuracy for the grouped condition (compatible distractor: *M* = 62.2%, *SD* = 18%; incompatible distractor: *M* = 56.8%, *SD* = 18%), *t*(37) = 2.28, *p* = .029, *g* = 0.30, and in the ungrouped condition (compatible distractor: *M* = 68.6%, *SD* = 20.2%; incompatible distractor: *M* = 58.6%, *SD* = 20.3%), *t*(37) = 3.92, *p* = .00037, *g* = 0.49 (see Fig. 3a). A 2 (grouped condition) × 2 (distractor type) repeated-measures ANOVA revealed a main effect for distractor type, *F*(1, 37) = 16.19, *p* = .00027, ${\eta}_p^2$ = 0.30, indicative of a significant flanker effect. There was also a marginal main effect for grouping condition, *F*(1, 37) = 3.99, *p* = .053, ${\eta}_p^2$ = 0.10, given overall higher performance when the target and distractor were not linked together. However, the size of the flanker effect did not depend on grouping condition, *F*(1, 37) = 2.19, *p* = .15, ${\eta}_p^2$ = 0.06.

This result is largely consistent with what we observed in Experiment 1, and a mixed-design comparison aligned with this observation. A 2 (large inducer vs. no inducer) × 2 (grouping condition) ANOVA failed to produce a significant main effect of inducer condition, *F*(1, 76) = 0.064, *p* = 0.80, ${\eta}_p^2$ = 0.00084*,* indicating that flanker effects collapsed across grouping condition were well matched between Experiment 1 and the large inducer blocks of Experiment 2. Similarly, when collapsing across experiments, there was no main effect of grouping condition, *F*(1, 76) = 2.88, *p* = 0.094, ${\eta}_p^2$ = 0.037. Most tellingly, the two factors did not interact, *F*(1, 76) = 0.69, *p* = .41, ${\eta}_p^2$ = 0.009. Thus, the patterns across grouping conditions did not depend on whether the flanker task was intermixed with a large inducer task or no inducer task at all.

##### Reaction time

One additional participant was removed from the RT analyses because they had no correct responses in one condition. As in Experiment 1, the RT data indicated that the accuracy results described above were not the product of a speed–accuracy trade-off. RTs were faster for compatible trials (*M* = 803 ms, *SD* = 202 ms) than incompatible trials (*M* = 854 ms, *SD* = 221 ms) across grouping conditions, and these were significantly different from each other, *F*(1, 36) = 10.96, *p* = .0021, ${\eta}_p^2$ = 0.23. As in Experiment 1, there was no main effect of grouping condition, *F*(1, 36) = 0.46, *p* = .50, ${\eta}_p^2$ = 0.013, nor was there a significant interaction between grouping condition and distractor type, *F*(1, 36) = 0.09, *p* = .77, ${\eta}_p^2$ = 0.0025. The RT data therefore mirrors the pattern observed in accuracy (see Fig. 3b).

#### Focal spotlight (small inducer blocks)

##### Accuracy

On small inducer blocks, we observed a main effect for distractor type, *F*(1, 37) = 6.94, *p* = .012, ${\eta}_p^2$ = 0.16, but not for grouping condition, *F*(1, 37) = 0.11, *p* = .74, ${\eta}_p^2$ = 0.0031. Departing from the results during a diffuse attentional state, however, here we observed a significant interaction between factors, *F*(1, 37) = 4.18, *p* = .048, ${\eta}_p^2$ = 0.10. When participants were induced into a focal attentional state around the target, a significant flanker effect again emerged within accuracy for the grouped condition (compatible distractor: *M* = 67.6%, *SD* = 21.6%; incompatible distractor: *M* = 57.5%, *SD* = 21.6%), *t*(37) = 3.32, *p* = .002, *g* = 0.46, but not for the ungrouped condition (compatible distractor: *M* = 65.2%, *SD* = 21.8%; incompatible distractor: *M* = 61.6%, *SD* = 19.8%), *t*(37) = 1.21, *p* = .24, *g* = 0.17 (see Fig. 3c).

We submitted the flanker effects from the small inducer blocks and Experiment 1 to a 2 (small inducer vs. no inducer) × 2 (grouping condition) mixed-design ANOVA. There was no main effect of inducer condition, *F*(1, 76) = 0.24, *p* = .62, ${\eta}_p^2$ = 0.0032*,* nor of grouping condition, *F*(1, 76) = 1.73, *p* = .19, ${\eta}_p^2$ = 0.022. However, we observed a small but significant interaction between factors, *F*(1, 76) = 4.76, *p* = .032, ${\eta}_p^2$ = 0.059. This suggests that the grouping condition differentially modulated the size of the flanker effect between Experiment 1 (no inducer task) and the small inducer blocks in Experiment 2.

##### Reaction time

One additional participant was removed from the RT analyses because they had no correct responses in one condition. RTs were well matched across conditions (grouped, compatible distractor: *M* = 777 ms, *SD* = 246 ms; grouped, incompatible distractor: *M* = 794 ms, *SD* = 216 ms; ungrouped, compatible distractor: *M* = 756 ms, *SD* = 257 ms; ungrouped, incompatible distractor: *M* = 768 ms, *SD* = 259 ms), and no comparisons produced significant differences. We did not observe a reliable main effect of grouping condition, *F*(1, 36) = 1.36, *p* = .25, ${\eta}_p^2$ = 0.036, nor of distractor type, *F*(1, 36) = 0.78, *p* = .38, ${\eta}_p^2$ = 0.021, nor an interaction, *F*(1, 36) = 0.044, *p* = .84, ${\eta}_p^2$ = 0.0012. Thus, once again, the patterns observed within percentage correct are not the result of a speed–accuracy trade-off (see Fig. 3d).

#### Assessing the relationship between inducer and flanker tasks

We assume the inducer task elicits sustained attention of a prescribed shape, size, and position that carries over to the intermixed flanker task, such that it may modulate the presence of an observable automatic OBA effect. The significant interaction between inducer size and grouping condition suggests this is the case. To further corroborate this assumption, we tested for significant relationships between the two tasks. We separately correlated accuracy on the large and small inducer tasks with OBA magnitude (defined as differential flanker performance between grouping conditions). A participant-level Pearson correlation between large inducer performance and OBA magnitude within the same blocks revealed no such relationship, *r* = .22, *t*(36) = 1.35, *p* = .18, suggesting that participants’ susceptibility to object contours (or lack thereof) did not depend on their engagement with the large inducer task. However, a significant negative relationship emerged for the same comparison on small inducer blocks, *r* = −.39, *t*(36) = −2.54, *p* = .015, and the two correlation coefficients differed significantly, *z* = 2.66, *p* =.0078. The differential patterns demonstrate that the correlations are not simply capturing general task proficiency, while the significant relationship on small inducer trials provides evidence of carryover effects.

Using a modified flanker task, we designed a paradigm where an ideal observer should attend only to the relevant portion of the stimulus display while ignoring the irrelevant portion, and where the two portions were sometimes grouped into a single object. To elicit sustained attention throughout the experimental session of a predictable size, shape, and position, we interleaved the flanker trials with more frequent inducer trials (Goodhew et al., 2016; Goodhew et al., 2017; Lawrence et al., 2020).

Here, we observed a significantly stronger flanker effect within accuracy in the grouped (compared to ungrouped) condition only when participants were concurrently engaged in the frequent small inducer task. The results indicate that an automatic spread of spatial selection from a relevant location to a grouped irrelevant location (Kramer & Jacobson, 1991) may only be detected when the observer has initially deployed a focal spotlight of spatial attention (Al-Janabi & Greenberg, 2016). The reliable interaction during periods of focused—but not diffused—selection indicates that in some circumstances, object contours can elicit an involuntary spread of selection that compete with both the task goals and current attentional state of the observer.

As indicated above, the pattern of results in Experiment 1 is most consistent with the large inducer blocks of Experiment 2. This conclusion is supported by the between-subjects ANOVA, which revealed no significant differences in flanker effects between Experiment 1 and large inducer blocks of Experiment 2. Furthermore, a nonsignificant correlation revealed that engagement with the large inducer task had no bearing on the magnitude of OBA effects, which significantly departed from the analogous analysis for small inducer blocks. Together, these results suggest that in the absence of a strong manipulation, a preponderance of participants spontaneously deployed relatively diffuse attention during the flanker task.

Importantly, the flanker task results from the small inducer condition of Experiment 2 rules out the possibility that, in the absence of any sustained attention manipulation, participants invariably albeit imperfectly ignored irrelevant space of an attended object. In contrast to an explanation invoking physiological limits, this pattern of results suggests that suppressing information within the irrelevant ring is overly effortful. Participants were unable to either derive or sustain an advantageously narrow spatial attention spotlight for the full length of the trial.

As noted in the Methods, a programming error led to a perfect correlation between object location (upper left vs. upper right quadrant) and distractor type (congruent vs. incongruent) that, had they noticed, participants could have exploited (e.g., by exerting greater suppressive effects of irrelevant space when an incongruent distractor is expected, given the location of the object display). We suspect that elements of the experimental design prevented participants from learning the relationship between location and distractor type, given that (1) flanker trials only made up one-third of the total trials, and (2) the target and distractor were only presented for 30 ms.

The data further supports our expectations. Because the error was consistent across all condition cells (grouping × inducer conditions), we would expect strategic exploitation to result in consistently weak or absent flanker effects. At worst, in the event the error was to have observable and disproportionate impacts on performance between conditions, we would wager a priori the impact should occur within large inducer blocks given the relatively wider attentional scope, potentially leading observers to notice the unwarranted coordination. This was not the case, however: An absent flanker effect only occurred in the ungrouped condition on small inducer blocks, while we observed significant flanker effects on large inducer blocks that did not differ from Experiment 1 (where no such programming error occurred).

A limitation of behavioral inducer studies, including this one, is that any changes in sustained attention artificially created by the inducer task cannot be directly measured (Lawrence et al., 2020). Instead, we assume that participants were actively engaged in the inducer task and attentional deployment on these trials carried over to the intermixed and less frequent flanker trials. We have indirect evidence to indicate this is likely the case: The flanker effect was statistically absent in the ungrouped condition during focal attention blocks, suggesting attention was effectively restricted to the relevant ring. This contrasts with the significant flanker effects that were observed in the same condition both under sustained diffuse attention and when attentional state was not experimentally manipulated (Experiment 1). This assumption is further supported by the subject-level correlation between small inducer performance and object-based selection within the same blocks: Participants’ susceptibility to the grouping condition depended on their accuracy on the inducer task.

Notably, the correlation for small inducer blocks revealed a negative relationship, indicating that participants who struggled more tended to exhibit automatic object-based selection effects at numerically larger magnitudes than their better performing counterparts. This result suggests that the object-based effects we observed are likely due to a relatively weaker, albeit still present, focal attention state that can be overwritten by the presence of common object contours extending beyond the attended region. It is possible that a stronger focal state (indicated by better small inducer performance) may mitigate this automatic selection. Given that performance on the small inducer task was well below ceiling, with all but one subject achieving scores of 85% or less, we cannot assess that here. It is, however, an intriguing hypothesis for future research.

## General discussion

The present study investigated whether cueing effects can result when sensory stimulation and task goals are opposed. Borrowing from the OBA literature—which has occasionally (albeit inconsistently) shown an involuntary object-based modulation of spatial selection—we presented target and distractor stimuli within either two independent or connected object frames. By combining an object-based display manipulation with a modified flanker task, we explicitly tested whether the attentional spotlight conforms to the contours of an object frame even when doing so is detrimental to performance.

Shomstein and Yantis (2004) observed robust OBA effects in a two-rectangle display only at short object-to-target SOAs (less than 400 ms), but no such effects at longer delays. As a result, the authors put forth a descriptive model of a brief, default mode of attention that relies on sensory processing for the purposes of object segmentation. This quickly gives way to a deliberate mode that emphasizes behavioral context. In our case, the long object display period ensured that object segmentation was fully resolved before target onset, and top-down attention fully deployed. Thus, rather than interrogating separable components of a serial process, we can investigate the role of object contours when they directly conflict with the observer’s current goal state. We furthermore manipulated the shape of sustained attention to reveal clear boundary conditions where trial-by-trial object-based effects may be expected in similar designs.

Although Experiment 1 failed to produce an object-based effect, a reliable one emerged in Experiment 2 when participants were induced to maintain a relatively focused attentional field around the relevant location. These patterns of results are largely consistent with previous literature (Experiment 2 of Al-Janabi & Greenberg, 2016; Shomstein & Yantis, 2002). Al-Janabi and Greenberg demonstrated when OBA was tested in weakly integrated displays (i.e., a distinct target encompassed by an object frame), the effects were absent when attention was presumably diffused and only present with an informative, spatially specific cue. Similarly, Shomstein and Yantis (2002) largely failed to detect OBA effects using a modified flanker task within a weak integration display in the absence of a cue. However, adding an uninformative spatial cue elicited OBA effects: Flanker interference was stronger when targets appeared on the spatially cued object than when they appeared at equidistant locations on an uncued object. Because the uninformative cue often necessitated space-based attentional shifts to locate the target, ultimately the authors interpreted the results as being consistent with a voluntary, attentional prioritization account of object-based selection.

Despite similar patterns of results across the current and previous studies, our experimental design precludes us from adopting a voluntary attention framework for our findings. Here, target certainty was perfectly predictable on a trial-by-trial basis, with the target always appearing in the ring closest to fixation. Given an appropriate selection of space during the 1,250-ms object display period, no additional attentional shifts were necessary upon target presentation. This indicates that attentional shifts to task-relevant locations is not a uniform precursor to object-mediated space-based selection. Instead, we argue that under certain circumstances, OBA may be involuntarily deployed, and that in such cases, the relationship previously observed between voluntary OBA and spatial attentional focus consistently holds.

The question remains, what circumstances should lead to involuntarily deployment? Lamy and Egeth (2002) employed a flanker experiment similar to the present study, but observed a different pattern of results. Using a fully valid spatial cue in a two-rectangle display, targets and compatible or incompatible distractors appeared either within the same or different rectangular frames. They did not observe a larger flanker effect in the grouped condition. The authors conclude, like others, that OBA deployment emerges only when attentional shifts are required.

Although we cannot definitively conclude why the current study departs from Lamy and Egeth (2002), we speculate on two possible factors. First, although each rectangular frame was presented in roughly the same parafoveal space as our relevant ring (4.4° from fixation), the boundaries of the irrelevant ring in our study extended further peripherally, possibly increasing the likelihood of involuntary spatial spread. Relatedly, Scolari and Awh (2019) suggested that feature integration is relatively high in the periphery, likely hindering individuation of distinct stimuli, and this is especially true with perceptually similar objects (Kimchi & Pirkner, 2015). Second, the two studies used qualitatively distinct cues to shape spatial selection: Lamy and Egeth used a traditional peripheral cue appearing after the object frames onset, while we used an inducer that participants engaged with during an orthogonal task on interspersed trials. Despite these design differences, both serve similar purposes and we are not the first to compare studies using similarly divergent cues (Lawrence et al., 2020). However, it is possible the inducer task by its nature more reliably elicits focal attention (in the case of the small inducer) of a prescribed shape. In sum, integration across both rings, regardless of grouping condition, may have contributed to the large flanker effects in Experiment 1. In Experiment 2, the focal spotlight condition effectively mitigated this undue spatial integration only when the rings were ungrouped.

Similarly, Al-Janabi and Greenberg (2016) failed to find an OBA effect when they used an uninformative peripheral cue, despite observing a robust one using a predictive cue (75% valid) in the same weakly integrated display. Given that the two experimental designs differed only by the validity of the cue, the authors concluded that neither perfect target certainty nor complete uncertainty should elicit strategically deployed object-based selection. In contrast, our account would suggest that, within certain sensory conditions, automatic OBA can occur regardless of cue validity. In light of this discrepancy, we consider a possible alternative explanation for the absent OBA effect presented in Al-Janabi and Greenberg. Although cueing effects were present in both experiments, the spatial validity effect within RT was roughly 2.5 times larger following an informative peripheral cue than an uninformative peripheral cue (126 ms vs. 51 ms). This leaves open the possibility that participants did not attend as strongly to the cued location following the uninformative, exogenous cue or that it did not elicit as narrow of an attentional focus—where both Al-Janabi and Greenberg and the current study demonstrate the necessity of narrowed focus to observe OBA effects in weakly integrated displays. In fact, as described in our previous discussion of Shomstein and Yantis (2002), it is possible to observe OBA effects even with an uninformative spatial cue.

Importantly, our conclusions do not rule out or detract from voluntary accounts of OBA, nor do we intend to call for a large-scale reinterpretation of previous studies. Rather than OBA being entirely automatic or entirely obligatory, we expect the truth is more nuanced and heavily dependent on task design. We suspect that a significant factor is a balance of effort to elicit adequate performance: Where selection of space via object contours is more effortful than suppressing them, full object selection may be voluntarily controlled; where circumstances lead to the reverse, full object selection may occur automatically. Scolari et al. (2014) made a similar proposal, suggesting that object-based selection occurs automatically when decomposing an object into relevant and irrelevant components is difficult. As described above, this is likely the case with more peripheral stimuli.

Thus far, we have suggested participants may have broadly attended to both rings—or otherwise failed to suppress the irrelevant ring—during Experiment 1. This is supported by large flanker effects across grouping conditions, as well as pattern similarities between Experiment 1 and the diffuse spotlight condition in Experiment 2. An alternative account for the data, however, is that attention was initially distributed focally to the relevant ring throughout the long SOA, but the sudden onset of the peripheral distractor captured attention (Barras & Kerzel, 2017; Ling & Carrasco, 2006; Yantis & Jonides, 1990) to produce a transient widened scope. Although plausible, we have several reasons to suspect that this is not the case. First, the 30-ms stimulus presentation is too short to elicit an attentional shift before offset (Ling & Carrasco, 2006; Müller & Rabbitt, 1989). Second, based on this account, we would expect attention to remain focused on no distractor trials in Experiment 1—given the absence of a sudden peripheral onset during target presentation—resulting in comparatively better performance. Instead, we observed no difference in performance between no distractor and compatible distractor trials, indicating attention was uniformly distributed across both trial types. Finally, when we experimentally induced a focal spotlight in Experiment 2, the sudden onset of peripheral distractors did not seemingly capture attention in the ungrouped condition, as indicated by the absent flanker effect. Thus, the sudden onset of the distractors alone unlikely account for our patterns of results.

The results across both experiments presented here indicate the contours of an object can shape spatial selection, even when such selection conflicts with behavioral goals. That this does not occur uniformly in the literature leads us to conclude that such automatic selection depends on task design and the requisite effort needed to suppress such selection (Scholl et al., 2001; Scolari et al., 2014).

Space-based attention mechanisms involve both enhancing the relevant signal and suppressing irrelevant signal—often simultaneously (Carrasco, 2011). Scolari and Awh (2019) compared performance on interference-absent and interference-present displays in conjunction with an uninformative spatial cue to isolate contributions of enhancement and suppression. Across six experiments, the results demonstrated that external noise suppression occurs in addition to signal enhancement when distinguishable distractors are present. We cannot disentangle the two processes with the current experimental design. As such, we have generally described flanker interference to be indicative of undue enhancement, failed suppression, or both. Similarly, the absent flanker effect observed in the ungrouped condition during small inducer blocks is equally consistent with a narrowed attentional focus and/or adequate suppression of irrelevant space. Future research may include a trial-by-trial cue validity manipulation to tease these attentional contributions apart.

Recently, the use of inducer tasks with unfilled objects has been criticized, as participants may focus on single aspects of the object (such as the edges), without eliciting a spatial spread throughout the enclosed space (Lawrence et al., 2020). For example, previous studies (Goodhew et al., 2016; Goodhew et al., 2017) employed a small inducer that exactly matched the contours of the spatial discrimination task targets (while the large inducers encompassed a larger space), such that attending only to the contours could benefit performance on those trials. However, this is not a concern in the current study, as even the small inducer still extended beyond the boundaries of the flanker task target. Thus, if participants were only attending to the contours and not to the full extent of the inducer, we would not expect to see any impact of the inducer task on flanker performance. That we do is evidence that a spatial spread indeed occurred on these trials, which is consistent with demonstrations of object-mediated spatial selection (Chen & Cave, 2006, 2008; Egly et al., 1994; Fiebelkorn et al., 2013; Moore et al., 1998).

## Conclusion

We investigated whether spatial selection can be shaped by sensory input even when it is directly at odds with behavioral goals. Here, we found a previously observed relationship between attentional scope and voluntary object-mediated space-based selection holds for stimulus-driven object selection as well. However, in contrast to voluntary accounts, we argue that when display parameters elicit sufficient feature integration across spatial cues, attention may be naturally distributed to the full extent of the stimulus space. Inducing sustained, focal attention can mitigate this undue integration across disjoined space but may fail to do so when objecthood is strong (i.e., the same region is included in a single enclosed object). This study adds important nuance to the literature on voluntary versus involuntary accounts of object-mediated space-based selection.
